# Gonorrhœa of the Mucous Membrane of the Mouth of the New-Born

**Published:** 1892-01

**Authors:** 


### GONORRHŒA OF THE MUCOUS MEMBRANE OF THE MOUTH IN THE NEWBORN.

Five cases of gonorrhœal infection of the mouth in infants are reported by Rosinski in the *Zeitschrift fnr Geburtshulfe und Gynakologie*, Band xxii., Heft 1, 1891. In most of the cases the maternal source of infection was clearly demonstrated. The lesion was an infiltration of the tissues upon the tongue and hard palate, with a characteristic yellowish mass composed of gonococci and pus-cells. The general health of the children was not especially disturbed, and very little pain or distress was observed. In some instances, gonorrhœal opthalmia was also present. The children recovered under appropriate treatment. Those who had opthalmia were sickest, and suffered considerably.—*Amer. Jour. Med. Sciences.*

